# Designing the Ideotype Mycorrhizal Symbionts for the Production of Healthy Food

**DOI:** 10.3389/fpls.2018.01089

**Published:** 2018-08-14

**Authors:** Luciano Avio, Alessandra Turrini, Manuela Giovannetti, Cristiana Sbrana

**Affiliations:** ^1^Department of Agriculture, Food and Environment, University of Pisa, Pisa, Italy; ^2^Interdepartmental Research Center “Nutraceuticals and Food for Health”, University of Pisa, Pisa, Italy; ^3^Institute of Agricultural Biology and Biotechnology, C.N.R., UOS Pisa, Pisa, Italy

**Keywords:** arbuscular mycorrhizal symbionts, healthy food, nutraceutical value, sustainable agriculture, secondary metabolism gene regulation, AMF functional diversity, health-promoting phytochemicals

## Abstract

The new paradigm in agriculture, sustainable intensification, is focusing back onto beneficial soil microorganisms, for the role played in reducing the input of chemical fertilizers and pesticides and improving plant nutrition and health. Worldwide, more and more attention is deserved to arbuscular mycorrhizal fungi (AMF), which establish symbioses with the roots of most land plants and facilitate plant nutrient uptake, by means of a large network of extraradical hyphae spreading from colonized roots to the surrounding soil and functioning as a supplementary absorbing system. AMF protect plants from biotic and abiotic stresses and are able to modulate the activity of antioxidant enzymes and the biosynthesis of secondary metabolites (phytochemicals), such as polyphenols, anthocyanins, phytoestrogens and carotenoids, that play a fundamental role in promoting human health. An increasing number of studies focused on the use of AMF symbionts for the production of functional food, with enhanced nutritional and nutraceutical value. Yet, while several plant species were investigated, only few AMF were utilized, thus limiting the full exploitation of their wide physiological and genetic diversity. Here, we will focus on AMF effects on the biosynthesis of plant secondary metabolites with health-promoting activity, and on the criteria for a finely tuned, targeted selection of the best performing symbionts, to be utilized as sustainable biotechnological tools for the production of safe and healthy plant foods.

## Introduction

The new paradigm in agriculture, sustainable intensification, is focusing back onto beneficial soil microorganisms, for the role played in reducing the input of chemical fertilizers and pesticides, while improving plant nutrition and health (Philippot et al., 2013). Worldwide, more and more attention is deserved to arbuscular mycorrhizal (AM) fungi (AMF), a key functional group of beneficial soil microbes belonging to the subphylum Glomeromycotina (Spatafora et al., 2016), able to establish a mutualistic symbiosis with the roots of 80% of plant species (Smith and Read, 2008). These encompass the most important crops for human consumption, such as wheat, rice, corn, barley, pulses, oats and millet, grapevine, olive, vegetables like strawberries, potato, tomato, medicinal plants and economically important species, such as sunflower, sugarcane, cotton, tobacco, coffee, tea, cocoa, rubber and cassava. AMF do not show host specificity, but are asexual obligate biotrophs, unable to complete their life cycle without host plants.

AMF life cycle is simple: germinating spores originate a short-lived mycelium able to recognize the roots of host plants and to differentiate appressoria on their surface. Then appressoria produce hyphae growing intercellularly in the roots and arbuscules, a sort of haustoria formed within root cells, where nutrient exchanges between the two symbionts occur. Up to 20% of total plant photosynthates is transferred to AMF, which, as chemoheterotrophs, utilize them as carbon source (Jakobsen et al., 1992; Smith and Read, 2008; Giovannetti et al., 2012); such transfer enables AMF to grow and form new spores. On the other hand, the extensive extraradical mycelium (ERM) explores the surrounding soil beyond the depletion zone around roots, and increases the root absorbing surface (up to 40 times) (Giovannetti et al., 2001). ERM is able to uptake and translocate soil mineral nutrients, such as phosphorus (P), nitrogen (N), sulfur, potassium, calcium, iron, copper, and zinc, thus improving plant growth and biomass production (Lehmann and Rillig, 2015). In addition, AMF provide diverse ecosystem services, enhancing water uptake, and increasing plant tolerance to biotic and abiotic stresses (Gianinazzi et al., 2010), thereby decreasing the need of chemical fertilizers and pesticides inputs (Toussaint et al., 2008; Sikes et al., 2009).

Several studies reported that AMF may modulate the synthesis of secondary metabolites in host plants, leading to a higher activity of antioxidant enzymes and enhancing the levels of diverse phytochemicals with health-promoting activities (Sbrana et al., 2014). Such findings are very important, as worldwide both consumers and producers are increasingly interested in the health-promoting properties of plant-derived foods. Indeed a number of epidemiological studies reported the role played by some plant secondary metabolites, including polyphenols, glucosinolates, flavonoids and carotenoids in the prevention of chronic diseases, arteriosclerosis, heart diseases and cancer (Duthie, 2000; Johnson, 2002; Lund, 2003). For example, theaflavins and thearubigins from black teas showed antiproliferative action (Bhattacharya et al., 2009), grape seed extract exerted preventive effects against human colon carcinoma and lung epithelial cancer (Wang et al., 2007; Lazzè et al., 2009), luteolin, kaempherol, apigenin and myricetin from diverse fruits and vegetables possessed anti-inflammatory and antibacterial activities (Dillard and German, 2000) and essential oils from myrtle displayed antimutagenic and antigenotoxic properties (Mimica-Dukić et al., 2010). In addition, glucosinolates from broccoli, cauliflower and cabbage were able to modulate carcinogens metabolism and detoxification (Dillard and German, 2000; Tang et al., 2010), while allicin and its organosulfur derivatives from garlic showed antitumoral activities in diverse human cancers (Butt et al., 2009; Teiten et al., 2013; Zhang et al., 2015). Although such phytochemicals are expressed mainly depending on plant genotype, their production may be modulated by diverse agronomic and environmental factors, including AMF symbioses. Here, we will focus on AMF ability to modulate the biosynthesis of plant secondary metabolites with health-promoting activity, and on the criteria for a finely tuned, targeted selection of the best performing symbionts, to be utilized as sustainable biotechnological tools for the production of safe and healthy plant foods.

## The production of phytochemicals by mycorrhizal plants

A large body of evidence showed that the establishment of AM symbiosis induces changes in plant physiology, modulating the activity of host cell primary and secondary metabolism (Fester and Hause, 2005; Lohse et al., 2005; Schliemann et al., 2008; Wipf et al., 2014; Schweiger and Müller, 2015; Cervantes-Gámez et al., 2016). Many authors investigated the changes induced by AMF in secondary metabolism, in relation to the production of functional compounds in roots, shoots, leaves, fruits and seeds of many different plant species (Sbrana et al., 2014).

Mycorrhizal plants produced higher amounts of phytochemicals with therapeutic value, such as the phytoestrogens biochanin A, formononetin, genistein, daidzein, showing a preventive action in osteoporosis, menopausal symptoms and degenerative diseases (Ososki and Kennelly, 2003; Khaosaad et al., 2008), sesquiterpene lactones, able to inhibit cell proliferation and tumor growth (Jurkiewicz et al., 2010; Teiten et al., 2013), the cardioactive and hypotensive alkaloid forskolin (Sailo and Bagyaraj, 2005), furanocoumarins (angelicin and psoralen) and the chemotherapeutic agents pterocarpans (erybraedin C and bitucarpin A), able to induce apoptosis in human colon carcinoma cell lines (Maurich et al., 2006; Pistelli et al., 2017).

Different species of medicinal and aromatic plants were investigated for their phytochemical contents upon mycorrhizal colonization, showing higher shoot levels of antioxidant compounds, such as rosmarinic acid, caffeic acid and essential oils in basil (Copetta et al., 2006, 2007; Toussaint et al., 2008; Rasouli-Sadaghiani et al., 2010), and anthraquinone derivatives, such as hypericin and pseudohypericin in *Hypericum perforatum* (Zubek et al., 2012). Also the levels of essential oils showed altered profiles in mycorrhizal *Origanum* sp. (Karagiannidis et al., 2011), and large increases in the fruits of mycorrhizal *Coriandrum sativum, Anethum graveolens, Trachyspermum ammi*, in the leaves of *Artemisia annua* and in the seeds of *Foeniculum vulgare* (Kapoor et al., 2002a,b; Chaudhary et al., 2008). Moreover, mycorrhizal plants of *Stevia rebaudiana* showed enhanced levels of the health-promoting compounds steviol glycosides (Tavarini et al., 2018).

Apart from medicinal plants and herbs, works investigating the phytochemical content of mycorrhizal plants cultivated for human consumption encompass a limited number of species, like lettuce, onion, tomato, maize, artichoke, strawberry, pepper and sweet potato (Table 1). Most of the data available on edible plant products have been obtained by studying single plant varieties, while only few works investigated the differential responses of cultivars/varieties belonging to the same species of food plants. For example, different mycorrhizal strawberry varieties did not show comparable levels of anthocyanins, anthocyanidins and vitamin C in fruits, while only some green and red leaf lettuce varieties contained larger amounts of anthocyanins, carotenoids, chlorophylls, tocopherol, and total phenolics, and showed a higher antioxidant activity, compared with control plants (Table 1). This represents a limitation of the studies performed so far, given the large number of old and new varieties currently grown worldwide, which could be investigated and selected on the basis of their ability to produce beneficial compounds upon mycorrhizal inoculation. Such a selection would be particularly important for some vegetable species considered functional foods, i.e., globe artichoke, for its hepatoprotective, anticarcinogenic, antioxidative and antibacterial activities, and tomato, for its ability to reduce the risks of cancer and cardiovascular diseases (Canene-Adams et al., 2005). Indeed, artichoke and tomato showed higher antioxidant activity and enhanced levels of health-promoting compounds when produced by AMF-inoculated plants (Table 1).

**Table 1:** Secondary metabolites and antioxidant activities in mycorrhizal food plants.

**Plant species**	**Variety or cultivar**	**AMF species**	**Measured metabolites/antioxidant activity assay method**	**Effect of AMF inoculation**	**References**
**(A) GREENHOUSE OR MESOCOSM EXPERIMENTS**					
***Solanum lycopersicum*** **L**.					
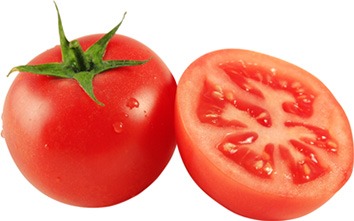	F1 Hybrid, GS-15	Mix of: *Glomus intraradices, Glomus mosseae, Glomus etunicatum* (Soil and Water Institute of Iran)	Lycopene (FW) DPPH^a^	Increased concentration Increased activity	Ordookhani et al., 2010
	Guadalete	Mix of: *Glomus mosseae, Glomus caledonium, Glomus viscosum, Glomus intraradices, Glomus coronatum*	Lycopene and β-carotene Lutein Ascorbic acid	No effect No effect Decreased concentration	Copetta et al., 2011
	Moneymaker	*Glomus intraradices* IMA6	Lycopene (FW) Total phenols (FW) Ascorbic acid (FW) Glutathione (FW) ABTS^b^ (FW)	Increased concentration No effect No effect No effect No effect	Giovannetti et al., 2012
	Nemo-Netta	*Glomus mosseae* (Biocult Ltd., South Africa)	Lycopene (FW) Total flavonoids (FW) Ascorbic acid (FW) ABTS	Increased concentration, only at late inoculation time No effect No effect No effect	Nzanza et al., 2012b
	Moneymaker	*Funneliformis mosseae* BEG12 and/or *Rhizophagus irregularis* BB-E (Agrauxine, F)	Lycopene (FW)	Mixed inoculation: no effect; single isolate inoculation: increased concentration	Hart et al., 2015
			β-carotene and total carotenoids (FW)	Mixed inoculation: increased concentration; single isolate inoculation: no effect	
			29 Odor-active volatile compounds	Distinct phytochemical profiles, but variable quantitative effects	
	Komeett	*Rhizophagus irregularis* (Premier Tech Inc., Canada)	DPPH (DW) Vitamins B1, B3, B5, B6, ascorbic acid (FW) Vitamin B6, ascorbic acid (DW) Total carotenoids (DW)	No effect No effect Decreased concentration Increased concentration	Hart et al., 2015
***Capsicum annuum*** **L**.					
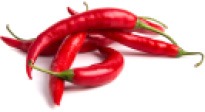	San Luis	*Glomus fasciculatum* or Mix1 (*Glomus constrictum, Glomus geosporum, Glomus fasciculatum, Glomus tortuosum*) or Mix2 (*Glomus aggregatum, Glomus deserticola, Glomus geosporum, Glomus microaggregatum, Sclerocystis coremioides*)	Carotenes Xanthophylls Capsaicinoids	Increased concentration by Mix2 Increased concentration by Mix2 No effect	Mena-Violante et al., 2006
	Cacho de cabra	*Glomus intraradices* (commercial) *Glomus claroideum* (native)	Ascorbic acid (FW)	Increased concentration (native), no effect (commercial)	Castillo et al., 2009
***Fragaria*** **x** ***ananassa*** **Duch**.					
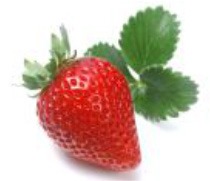	Aromas	*Glomus intraradices* (Premier Tech Biotechnologies Company, Canada)	Total phenols (DW) p-coumaric acid (DW) Gallic, ferulic, ellagic acids (DW) Cyanidin-3-glucoside (DW) Pelargonidin-3-glucoside (DW) Quercetin and kaempferol (DW) Catechin (DW)	No effect Increased concentration at intermediate N No effect or decreased concentration depending on N concentration Increased concentration at intermediate N No effect Increased concentration at intermediate N No effect	Castellanos Morales et al., 2010
	Selva	Mix (Mybasol, Italy)	Pelargonidin 3-glucoside, Pelargonidin 3-rutinoside and pelargonidin malonyl glucoside (FW) Pelargonidin acetyl glucoside and cyanidin 3-glucoside (FW) Total pelargonidins (FW)	Increased concentration No effect Increased concentration	Lingua et al., 2013
	Selva	Mix of: *Rhizophagus intraradices, Glomus aggregatum, Glomus viscosum, Claroideoglomus etunicatum, Claroideoglomus claroideum* (Mybasol)	Ascorbic acid (FW) Folate (FW)	Increased concentration No effect	Bona et al., 2015
	Fortuna Sabrina Splendor	*Glomus iranicum* var. *tenuihypharum* (Mycogrowth®, Spain)	Total phenols and Anthocyanins (FW) Ascorbic acid (FW) Total phenols, anthocyanins and ascorbic acid (FW) Total phenols and Ascorbic acid (FW) Anthocyanins (FW)	Increased concentration at early inoculation No effect No effect No effect Decreased concentration	Cecatto et al., 2016
***Lactuca sativa*** **L**.					
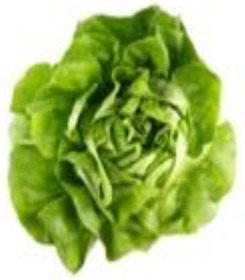	var. *capitata*, Batavia Rubia Munguía var. *capitata*, Maravilla de Verano	*Glomus fasciculatum*	Carotenoids (FW) Total phenols and ascorbic acid (FW) Anthocyanins (FW) Carotenoids, total phenols and ascorbic acid (FW) Anthocyanins (FW)	Increased concentration depending on leaf position No effect Increased concentration No effect Increased concentration	Baslam et al., 2011
	var. *longifolia*, Cogollos de Tudela	*Glomus fasciculatum* or Mix of: *Glomus intraradices, Glomus mosseae* (Atens, Spain)	Carotenoids (FW) Total phenols (FW) Anthocyanins (FW) Ascorbic acid (FW)	No effect Increased concentration in outer leaves Increased concentration in inner leaves Increased concentration in inner leaves by *G. fasciculatum*	
	Batavia Rubia Munguía	Mix of: *Rhizophagus intraradices, Funneliformis mosseae* (Atens)	Carotenoids (FW) Total phenols (FW) Anthocyanins (FW) Ascorbic acid (FW)	Decreased concentration in autumn in inner leaves and increased in spring No effect Increased concentration in winter and spring in inner leaves Increased concentration in winter and spring in outer leaves	Baslam et al., 2013
	Maravilla de Verano		Carotenoids (FW) Total phenols (FW) Anthocyanins (FW) Ascorbic acid (FW)	Increased concentration in winter and spring in inner leaves No effect Increased concentration in winter in outer leaves and spring in inner leaves No effect	
	Batavia Rubia Munguía and Maravilla de Verano	Mix of: *Rhizophagus intraradices, Funneliformis mosseae* (Atens)	Total carotenoids (DW)	Increased concentration in Batavia Rubia Munguía	Goicoechea et al., 2015
			Epidermal flavonols Anthocyanins (by optical monitoring) Soluble phenols (DW) DPPH (DW)	Decreased levels in Batavia Rubia Munguía No effect No effect Increased activity in Batavia Rubia Munguía, decreased in Maravilla de Verano	
	not available	*Rhizophagus intraradices*	Superoxide dismutase and catalase Ascorbate peroxidase Glutathione reductase Total carotenoids (FW)	Increased activity Reduced activity No effect Increased concentration	Durán et al., 2016
	var. *crispa* Eluarde and Panisse	*Funneliformis mosseae* AZ225C or *Rhizoglomus irregulare* IMA6 (formerly *Glomus intraradices*)	ORAC^c^ (FW) Total phenolics (FW) Total anthocyanins (FW) (only Eluarde)	Increased activity Increased concentrations with IMA6 Increased concentrations	Avio et al., 2017
***Ocimum basilicum*** **L**.					
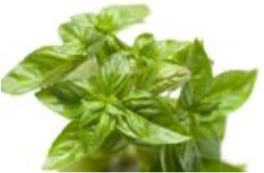	Genovese	*Glomus mosseae* BEG 12, or *Gigaspora margarita* BEG 34, or *Gigaspora rosea* BEG 9	Essential oils (13 terpenoids and 2 phenolic compounds)	Variable depending on AMF and compound	Copetta et al., 2006, 2007
	Genovese Italian and Purple Petra	*Glomus intraradices* (Native Plants Incorporated, USA)	Total anthocyanins (FW) Total phenolics and phenolic acids (FW)	Increased concentration in Purple Petra No effect	Lee and Scagel, 2009
	not available	*Glomus fasciculatum* or *Glomus etunicatum* or *Glomus intraradices*	Total essential oils (DW)	Increased concentration and distinct phytochemical profiles	Rasouli-Sadaghiani et al., 2010
	Cinnamon, Siam Queen, Sweet Dani and Red Rubin	*Rhizophagus intraradices* (Native Plants Incorporated)	Total anthocyanins (FW) Total phenolics (FW) Phenolic acids (FW) Total flavonoids (FW)	Increased concentration in Red Rubin No effect Increased concentration No effect	Scagel and Lee, 2012
	Tigullio and Dark Opal	*Rhizoglomus irregulare* IMA6 (formerly *Glomus intraradices*)	ABTS (DW) Total carotenoids (DW) Total phenolics (DW) Anthocyanins (DW) (Dark Opal) Rosmarinic acid (DW)	No effect Decreased concentration No effect Decreased concentration Decreased concentration	Battini et al., 2016b
***Cynara cardunculus*** **L. var**. ***scolymus***					
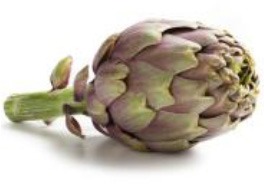	Terom	*Glomus mosseae* AZ 225C and/or *Glomus intraradices* IMA6	Total phenolics (FW) and DPPH	Increased concentration and activity with dual inoculation and *G.intraradices*	Ceccarelli et al., 2010
***Allium cepa L***.					
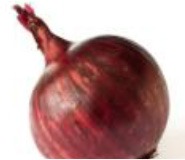	Centurion	Mix of: *Glomus mosseae, Glomus intraradices, Glomus claroideum, Glomus microaggregatum* (Plantworks, UK)	Organosulfur compounds (as total pyruvic acid) and total phenolics (DW) Quercetin monoglycoside (DW) Quercetin diglycoside (DW) ABTS and ESR^d^ (DW)	No effect Increased concentration when ${\text{NO}}_{3}^{-}$-N predominant No effect Increased activity when ${\text{NO}}_{3}^{-}$-N predominant	Perner et al., 2008
	Nasik red N-53	Mix of: *Glomus intraradices, Glomus mosseae*	Total phenolics (FW)	Increased concentration	Lone et al., 2015
	Alice	Mix of: *Glomus etunicatum, Glomus microaggregatum, Glomus intraradices, Glomus claroideum, Glomus mosseae, Glomus geosporum* (Symbivit, Czech Rep.) or *Glomus intraradices* BEG140	FRAP^e^ Ascorbic acid (FW)	Increased activity with Mix No effect	Albrechtova et al., 2012
	Stuttgarter Riesen	Mix of: *Funneliformis mosseae* and *Rhizophagus irregularis* (INOQ, Germany)	Quercetin-diglucoside and quercetin-monoglucoside Isorhamnetin-glucoside	Increased concentration at high inoculation amount and when ${\text{NH}}_{4}^{+}$-N predominant No effect	Mollavali et al., 2018
***Ipomea batatas*** **L**.					
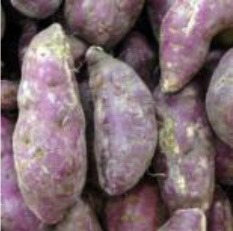	not available	*Glomus intraradices* (IFP Glintra, INOQ) and/or *Glomus mosseae* (IFP Glm, INOQ)	β-carotene (DW)	Increased concentration	Tong et al., 2013
**(B) FIELD EXPERIMENTS**					
***Solanum lycopersicum*** **L**.					
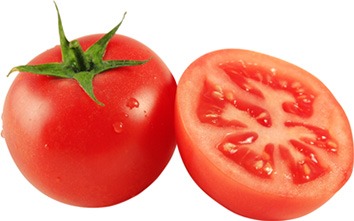	PKM-1	*Glomus intraradices* # TNAU 120-02	Ascorbic acid (FW)	Increased concentration	Subramanian et al., 2006
	Vitella F1	*Glomus* sp. (Amykor, Germany)	Lycopene (FW) β-carotene and Total phenols (FW)	Increased concentration Increased concentration, under organic management	Ulrichs et al., 2008
	Nemo-Netta	*Glomus mosseae* (Biocult Ltd., South Africa)	Ascorbic acid (FW)	Increased concentration	Nzanza et al., 2012a
	TC 2000	Mix of: *Rhizophagus intraradices, Glomus aggregatum, Glomus viscosum, Claroideoglomus etunicatum* and *Claroideoglomus claroideum* (Mybasol, Italy)	Lycopene β-carotene (FW) Ascorbic acid (FW)	No effect No effect Decreased concentration	Bona et al., 2017
	Perfect Peel, Roma, Rio Grande	Mix of*: Rhizoglomus irregulare* IMA6 (formerly *Glomus intraradices*)*, Funneliformis mosseae* IMA1	Lycopene (FW)	No effect	Njeru et al., 2017
***Cynara cardunculus*** **L. var**. ***scolymus***					
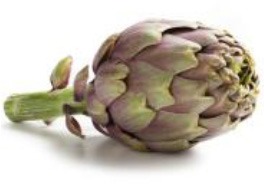	Terom	*Glomus mosseae* AZ 225C and/or *Glomus intraradices* IMA6	Total phenolics (FW) DPPH	Increased concentration Increased activity, only with dual inoculation in the second year	Ceccarelli et al., 2010
	Romanesco' type cv. C3	Mix of: *Glomus mosseae, Glomus intraradices* (Italpollina, Italy) (Aeg) or *Glomus intraradices* (Tecnologiás Naturales Internacional, Mexico) (End)	ABTS (DW) Total phenolics (DW)	No effect Increased concentration with End; variation in phenolic acids and flavonoid profiles	Palermo et al., 2013
	Violetto di Sicilia	*Glomus viscosum* (syn. *Septoglomus viscosum*)	Caffeoylquinic acids, apigenin and luteolin (DW)	Increased concentration in receptacles, compared to traditional vegetative reproduced plants	Pandino et al., 2017
	Romolo and Istar	Mix of: *Rhizophagus intraradices* BEG72, *Funneliformis mosseae* (Italpollina, Italy)	Total phenolics (DW) ABTS (DW) DPPH (DW)	Increased concentration in primary heads, decreased in secondary heads Increased activities Increased activities in primary heads, no effect or reduced activity in secondary heads depending on cultivar	Rouphael et al., 2017
***Allium cepa*** **L**.					
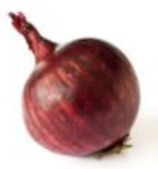	Hyskin	Vaminoc (MicroBio, UK) or *Glomus intraradices* BEG87	Quercetin (FW)	No effect	Mogren et al., 2007
***Ipomea batatas*** **L**.					
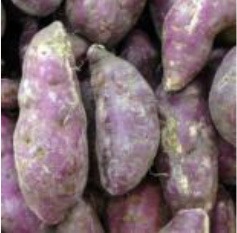	Hongxin	*Glomus etunicatum* BEG 168, *Glomus etunicatum* HB-Bd45-Gsp4, *Glomus intraradices* BEG 141, and a mix of them (M3); *Glomus mosseae* BEG 167, a mix of M3 and BEG 167 (M4*)*; mix of : *Glomus intraradices* and *Glomus mosseae* (Biorize, France)	Carotene	No effect	Farmer et al., 2007

*Data are reported on a fresh weight (FW) or dry weight (DW) basis; where unspecified, no information was available. The binomial nomenclature of AMF reported in the quoted papers has been maintained*.

a *DPPH, 2,2-Diphenyl-1-picrylhydrazyl assay*;

b *ABTS, 2,2'-azino-bis(3-ethylbenzothiazoline-6-sulphonic acid) assay*;

c *ORAC, oxygen radical absorbance capacity assay*;

d *ESR, electron spin resonance spectroscopy*;

e *FRAP, ferric reducing ability of plasma assay*.

The mechanistic explanation of the differential biosynthesis of secondary metabolites in mycorrhizal plants involves the activity of diverse enzymes leading to the production of terpenoids, flavonoids and the aminoacids tyrosine and phenylalanine, precursors of polyphenols in the phenylpropanoid pathway (Peipp et al., 1997; Walter et al., 2000; Lambais et al., 2003; Ponce et al., 2004; Herre et al., 2007; Pozo and Azcon-Aguilar, 2007; López-Ráez et al., 2010a). Such molecules, often accumulated in plant resistance reaction to biotic and abiotic stresses, may be modulated by plant hormones such as ABA or jasmonates possibly involved in long distance signaling and in mycorrhizal priming of defense responses (Cameron et al., 2013; Adolfsson et al., 2017). Several gene expression studies showed a differential modulation of genes encoding for key enzymes of biochemical pathways leading to the production of health-promoting secondary metabolites in food plants (Table 2) and model plant species (Harrison and Dixon, 1993, 1994; Bonanomi et al., 2001; Liu et al., 2007; Handa et al., 2015). In food plants, the use of the RNA-Seq technology, allowing investigations of whole transcripts, revealed that many genes, belonging to different functional classes, i.e., post-translational regulation, signaling, transport, biotic and abiotic stresses and hormone metabolism, were upregulated upon AMF inoculation and differentially expressed in fruits, leaves and roots, compared with controls (Table 2). Unfortunately, most of currently available RNA-Seq data assessing mycorrhizal regulated genes derive only from roots of the investigated plants, such as *Citrus sinensis, Cucumis sativus, Helianthus annuus, Litchi chinensis, Oryza sativa*, and *Vitis vinifera* (Table 2). Since the different genes may be differentially expressed in the diverse plant organs, further works should focus on the edible parts of food plants, in order to obtain information on the genes regulating the production of health-promoting compounds, modulated by mycorrhizal symbioses. In addition, the functional significance of fungal symbiont identity in the modulation of phytochemicals production should be deeply investigated, as large variations in gene expression were detected in model plants inoculated with different AMF (Burleigh et al., 2002; Hohnjec et al., 2005; Deguchi et al., 2007; Massoumou et al., 2007).

**Table 2:** Genes involved in the biosynthesis of health-promoting secondary metabolites, which are upregulated in above- or belowground cell tissues of mycorrhizal food plants.

**Plant**	**Effective AMF**	**Plant tissue**	**Assay method**	**Upregulated gene/enzyme family**	**Involved pathway**	**References**
*Citrus sinensis*	*Glomus versiforme* (BGC HUN02B)	Leaves	RNA-Seq	12-oxophytodienoate reductase Acyl-CoA oxidase Enoyl-CoA hydratase Jasmonate O-methyltransferase Linoleate 13S-lipoxygenase OPC-8:CoA ligase	Secondary metabolites biosynthesis	Gao et al., 2016
*Cucumis sativus*	*Funneliformis mosseae*	Leaves	RT-qPCR	Caffeoyl CoA 3-O-methyltransferase Cinnamyl alcohol dehydrogenase-like protein Cytochrome P450 C4H cinnamate-4-hydroxylase Phenylalanine ammonia-lyase	Phenylpropanoid biosynthesis	Chen et al., 2013
*Cucumis sativus*	*Rhizophagus irregularis* (PH5, formerly Glomus intraradices)	Roots	RNA-Seq	Allene oxide synthase 1 Cinnamyl alcohol dehydrogenase (6-like) Cytochrome P450 (734A6-like) Gibberellin 2-beta-dioxygenase	Secondary metabolites biosynthesis Phenylpropanoid biosynthesis Phenylpropanoid biosynthesis Diterpenoid biosynthesis	Ma et al., 2018
*Glycine max*	*Rhizophagus irregularis* (49, formerly Glomus intraradices)	Roots	Microarray	9-cis-epoxycarotenoid dioxygenase Cytochrome P450 monooxygenase (CYP 701) Geranylgeranyl diphosphate or pyrophosphate synthase Gibberellin 2-beta-dioxygenase Gibberellin 3-beta (20)-dioxygenase Isoflavone-O-methyltransferase Trihydroxyisoflavanone-O-methyltransferase Tropinone reductase	Carotenoid biosynthesis Terpenoid backbone biosynthesis Terpenoid backbone biosynthesis Diterpenoid biosynthesis Diterpenoid biosynthesis Isoflavonoid biosynthesis Isoflavonoid biosynthesis Alkaloids biosynthesis	Schaarschmidt et al., 2013
				Cytochrome p450 (CYP93A-like) Ent-kaurene oxidase cyp701a5 Flavanone 3-hydroxylase Flavonoid glucosyltransferase Flavonoid peroxidase 1 Geraniol 8-hydroxylase-like Gibberellin 2-beta-dioxygenase Gibberellin 3-beta (20)-dioxygenase Hydroxycinnamoyl transferase Hyoscyamine 6-dioxygenase Isoleucine N-monooxygenase Pelargonidin 3-o-(6-caffeoylglucoside) 5-o-(6-o-malonylglucoside) 4-malonyltransferase-like Shikimate o-hydroxycinnamoyltransferase-likeTetrahydrocannabinolic acid synthase-like Tropinone reductase homolog Valine N-monooxygenase (CYP79D1-2) Zeatin-O-xylosyltransferase-like	Isoflavonoid biosynthesis Diterpenoid biosynthesis Flavonoid biosynthesis Flavonoid biosynthesis Flavonoid biosynthesis Monoterpenoid biosynthesis Diterpenoid biosynthesis Diterpenoid biosynthesis Phenylpropanoid biosynthesis Alkaloid biosynthesis Glucosinolate biosynthesis Anthocyanin biosynthesis Flavonoid biosynthesis Cannabinoid biosynthesis Alkaloids biosynthesis Glucosinolate biosynthesis Secondary metabolites biosynthesis	Vangelisti et al., 2018
*Litchi chinensis*	native AMF community	Roots	RNA-Seq	Anthocyanidin reductase Bifunctional dihydroflavonol 4-reductase/flavanone 4-reductase Caffeic acid 3-O-methyltransferase Carotenoid cleavage dioxygenase 7 Chalcone synthase Coumarate-CoA ligase 2 Cytochrome P450 (CYP73A) Flavanone 3-dioxygenase Flavonoid 3′-monooxygenase Leucoanthocyanidin reductase Naringenin,2-oxoglutarate 3-dioxygenase Peroxidase 53 Tropinone reductase	Flavonoid biosynthesis Flavonoid biosynthesis Phenylpropanoid biosynthesis Carotenoid biosynthesis Flavonoid biosynthesis Phenylpropanoid biosynthesis Phenylpropanoid biosynthesis Flavonoid biosynthesis Flavonoid, flavone, flavonol biosynthesis Flavonoid biosynthesis Flavonoid biosynthesis Phenylpropanoid biosynthesis Alkaloid biosynthesis	Shu et al., 2016
*Ocimum basilicum*	*Rhizoglomus irregulare* (IMA6, formerly *Glomus intraradices*)	Leaves	RT-qPCR	Tyrosine amino-transferase	Tyrosine metabolism	Battini et al., 2016a
*Oryza sativa*	*Glomus intraradices* (DAOM197198)	Leaves	Macroarray	Ascorbate peroxidase (APX8) Cytochrome P450 Dehydroascorbate reductase Squalene monooxygenase	Ascorbate and aldarate metabolism Phenylpropanoid biosynthesis Ascorbate and aldarate metabolism Secondary metabolites biosynthesis	Campos-Soriano et al., 2012
	*Rhizophagus irregularis* (DAOM197198)	Roots	RNA-Seq	Anthocyanidin 3-O-glucosyltransferase Cinnamoyl-CoA reductase cytochrome P450 Ent-kaurene synthase Flavonol synthase/flavanone 3-hydroxylase Laccase precursor protein Mannose-6-phosphate isomerase Oxidoreductase, aldo/keto reductase family protein Phytoene synthase Terpene synthase	Anthocyanin biosynthesis Phenylpropanoid biosynthesis Phenylpropanoid biosynthesis Diterpenoid biosynthesis Flavonoid biosynthesis Ascorbate and aldarate metabolism Ascorbate and aldarate metabolism Retinoic acid biosynthesis Carotenoid biosynthesis Diterpenoid biosynthesis	Fiorilli et al., 2015
	*Glomus intraradices*	Roots	Microarray	Cinnamoyl-CoA reductase 4-coumarate CoA ligase Cycloartenol synthase Cytochrome p450 Flavonoid 3′,5′-hydroxylase Geranylgeranyl diphosphate synthase	Phenylpropanoid biosynthesis Phenylpropanoid biosynthesis Secondary metabolites biosynthesis Phenylpropanoid biosynthesis Flavonoid biosynthesis Terpenoid backbone biosynthesis	Güimil et al., 2005
	*Rhizophagus irregularis*	Roots	Microarray	4-coumarate-CoA ligase Caffeoyl CoA 3-O-methyltransferase Gibberellin 2-beta-dioxygenase p-coumaroyl-CoA:caffeoyl-CoA 3-hydroxylase	Phenylpropanoid biosynthesis Phenylpropanoid biosynthesis Diterpenoid biosynthesis Phenylpropanoid biosynthesis	Gutjahr et al., 2015
*Pisum sativum*	*Glomus mosseae* (BB-E-Sc-02; Biorize, Dijon, FR)	Roots	SSH	Beta-cyanoalanine synthase Neoxanthin (clavage enzyme) synthase	Secondary metabolites biosynthesis Carotenoid biosynthesis	Grunwald et al., 2004
*Solanum lycopersicum*	*Rhizophagus irregularis*	Leaves	RNA-Seq	4-coumarate CoA ligase Acyltransferase-like protein Caffeoyl CoA 3-O-methyltransferase Cinnamoyl CoA-reductase-like Cytochrome P450 NADPH-reductase Hydroxycinnamoyl transferase Hydroxycinnamoyl-CoA shikimate/quinate hydroxycinnamoyltransferase Mevalonate kinase Phenylalanine ammonia-lyase Undecaprenyl pyrophosphate synthase	Phenylpropanoid biosynthesis Phenylpropanoid biosynthesis Phenylpropanoid biosynthesis Flavonoid biosynthesis Phenylpropanoid biosynthesis Phenylpropanoid biosynthesis Phenylpropanoid biosynthesis Terpenoid backbone biosynthesis Phenylpropanoid biosynthesis Terpenoid backbone biosynthesis	Cervantes-Gámez et al., 2016
	*Glomus intraradices* (BEG141)	Roots	Microarray	Carotenoid cleavage dioxygenase Cytochrome P450 Mevalonate disphosphate decarboxylase Phytoene dehydrogenase	Carotenoid biosynthesis Phenylpropanoid biosynthesis Carotenoid biosynthesis Carotenoid biosynthesis	Dermatsev et al., 2010
	*Glomus mosseae* (BEG12)	Roots	RNA-Seq	(−)-a-terpineol synthase (+)-delta-cadinene synthase 4-coumarate CoA ligase Anthocyanidin synthase Carotenoid cleavage dioxygenase 1 Cytochrome P450 Cytochrome p450 monooxygenase cyp72a59 Monoterpene glucosyltransferase Phenylalanine ammonia-lyase Phytoene synthase Sesquiterpene synthase	Terpenoid backbone biosynthesis Terpenoid backbone biosynthesis Phenylpropanoid biosynthesis Flavonoid biosynthesis Carotenoid biosynthesis Phenylpropanoid biosynthesis Phenylpropanoid biosynthesis Terpenoid backbone biosynthesis Phenylpropanoid biosynthesis Carotenoid biosynthesis Terpenoid backbone biosynthesis	Fiorilli et al., 2009
	*Glomus mosseae* (BEG12)	Shoots	RNA-Seq	Caffeic acid 3-O-methyltransferase Cytochrome p450 Tropinone reductase	Phenylpropanoid biosynthesis Phenylpropanoid biosynthesis Alkaloid biosynthesis	
	*Glomus irregulare* (DAOM 197198)	Roots	Microarray	Allene oxide synthase 3 Gibberellin 3-beta-dioxygenase Phytoene desaturase	Secondary metabolites biosynthesis Carotenoid biosynthesis Carotenoid biosynthesis	Garrido et al., 2010
	*Glomus intraradices*	Roots	Microarray	Deoxy-D-xylulose-5-phosphate reductoisomerase Gibberellin 2-beta-dioxygenase Hydroxycinnamoyl/benzoyl transferase Lycopene b-cyclase Phytoene desaturase z-carotene desaturase	Terpenoid backbone biosynthesis Diterpenoid biosynthesis Phenylpropanoid biosynthesis Carotenoid biosynthesis Carotenoid biosynthesis Carotenoid biosynthesis	López-Ráez et al., 2010b
	*Glomus intraradices/Glomus mosseae*	Roots	Microarray	Allene oxide synthase 1-3 Antocyanin acyltrasferase Cytochrome P450 monooxygenase (CYP 81, CYP721) 1-deoxy-D-xylulose 5-phosphate synthase Gibberellin 3-beta (20)-dioxygenase	Secondary metabolites biosynthesis Anthocyanidin biosynthesis Terpenoid backbone biosynthesis Terpenoid backbone biosynthesis Diterpenoid biosynthesis	
	*Glomus mosseae*	Roots	Microarray	Polyphenol oxidase	Secondary metabolites biosynthesis	
	*Glomus mosseae*	Fruit	Microarray	Histidine decarboxylase	Secondary metabolites biosynthesis	Salvioli et al., 2012
	*Rhizophagus irregularis* (DAOM 197198)	Roots	RNA-Seq	Carotenoid cleavage dioxygenase 8 Cytochrome P450 (CYP93A1)	Carotenoid biosynthesis Flavonoid biosynthesis	Sugimura and Saito, 2017
	*Funneliformis mosseae* (BEG12)	Fruit	RNA-Seq	Terpene synthase	Diterpenoid biosynthesis	Zouari et al., 2014
*Solanum tuberosum*	*Glomus* sp. MUCL 41833	Roots	Microarray	Anthocyanidin-3-glucoside rhamnosyltransferase Anthranilate N-hydroxycinnamoyl/benzoyltransferase Benzyl alcohol benzoyl transferase Carotenoid isomerase Catechol oxidase Chalcone reductase Coumarate-CoA ligase (2-4) Cytochrome p450 (CYP71-like) Flavanone 3-hydroxylase Flavonoid 1-2 rhamnosyltransferase Flavonoid 3′-monooxygenase Geranylgeranyl pyrophosphate synthase 1 Gibberellin 2-beta-dioxygenase Hydroxycinnamoyl transferase Leucoanthocyanidin dioxygenase-like Orcinol O-methyltransferase Peroxidase (Class III) Phenylalanine ammonia-lyase Phytoene desaturase Hyoscyamine 6 beta-hydroxylase-likeSesquiterpene synthase 2 Tropinone reductase I Tyramine hydroxycinnamoyl transferase Vetispiradiene synthase z-carotene desaturase	Anthocyanin biosynthesis Secondary metabolites biosynthesis Phenylpropanoid biosynthesis Carotenoid biosynthesisIsoquinoline alkaloid biosynthesisFlavonoid biosynthesis Phenylpropanoid biosynthesis Phenylpropanoid biosynthesis Flavonoid biosynthesis Flavonoid biosynthesis Flavonoid, flavone, flavonol biosynthesis Terpenoid backbone biosynthesis Diterpenoid biosynthesis Phenylpropanoid biosynthesis Anthocyanidin biosynthesis Secondary metabolites biosynthesis Phenylpropanoid biosynthesis Phenylpropanoid biosynthesis Carotenoid biosynthesis Alkaloid biosynthesis Terpenoid backbone biosynthesis Alkaloids biosynthesis Phenylpropanoid biosynthesis Terpenoid backbone biosynthesis Carotenoid biosynthesis	Gallou et al., 2012
*Vitis vinifera*	*Funneliformis mosseae* (BEG12)	Roots	RNA-Seq	ABA 8′-hydroxylase CYP707A1 Alliin lyase precursor	Carotenoid biosynthesis Secondary metabolites biosynthesis	Balestrini et al., 2017
	*Funneliformis mosseae* (BEG12)/MICOSAT F® Vite (CCS-Aosta)	Roots	RNA-Seq	4-coumarate-CoA ligase ABA 8'-hydroxylase CYP707A1 Cinnamyl alcohol dehydrogenaseTaxane 10-beta-hydroxylase Tropinone reductase Urophorphyrin III methylase	Terpenoid-quinone biosynthesis Carotenoid biosynthesis Phenylpropanoid biosynthesis Carotenoid biosynthesis Alkaloids biosynthesis Secondary metabolites biosynthesis	
	MICOSAT F® Vite (CCS-Aosta)	Roots	RNA-Seq	Methyl jasmonate esterase	Secondary metabolites biosynthesis	
*Zea mays*	*Rhizophagus irregularis*	Leaves	Microarray	1-aminocyclopropane-1-carboxylate oxidase 3-hydroxy-3-methylglutaryl- reductase Alliin lyase	Cysteine and methionine metabolism Terpenoid backbone biosynthesis Secondary metabolites biosynthesis	Gerlach et al., 2015
				Anthocyanidin 3-O-glucosyltransferase 2 Beta-carotene hydroxylase 1 Cytochrome p450 71a1 Farnesyl pyrophosphate synthetase Gibberellin 2-beta-dioxygenase Homogentisate phytyltransferase vte2-1 Mevalonate kinase Short-chain dehydrogenase reductase family protein	Anthocyanin biosynthesis Carotenoid biosynthesis Phenylpropanoid biosynthesis Terpenoid backbone biosynthesis Diterpenoid biosynthesis Terpenoid-quinone biosynthesis Terpenoid backbone biosynthesis Retinoic acid biosynthesis	

*The binomial nomenclature of arbuscular mycorrhizal fungi (AMF) reported in the quoted papers has been maintained. Assay methods: RNA sequencing (RNA-Seq), quantitative reverse transcription PCR (RT-qPCR), suppression subtractive hybridization (SSH)*.

## Functional diversity of AMF

A number of studies have been carried out in order to select the optimal host/fungus combinations leading to the best plant performance, in terms of growth and nutrition. Different strategies have been proposed to detect the criteria for the selection of infective and efficient strains to be used for inoculation in diverse host plants and soil conditions. The possibility of implementing AMF inoculation depends first and foremost on the availability of strains able to establish rapidly an extensive colonization in the roots of a host plant and to compete for infection sites with indigenous AMF endophytes. Despite the obligately biotrophic status of AMF, involving labor-intensive experiments, progress has been done in the determination of the parameters that, in many different AMF isolate, affect spore dormancy and germination, pre-symbiotic mycelial growth, appressorium formation and intraradical development (Giovannetti et al., 2010). As to the selection of efficient isolates, the great majority of studies assessed their efficiency in terms of host growth responses, nutrient uptake, in particular P and N, and consequently considered the relevant fungal parameters, such as the extent and viability of ERM exploring the soil, the rate of absorption, translocation and transfer of mineral nutrients, from hyphae to plant root cells (Giovannetti and Avio, 2002; Ezawa and Saito, 2018).

Only few works studied the differential efficiency of the diverse AMF isolates in relation to the production of health-promoting phytochemicals. The first studies reported higher contents of essential oils in coriander shoots and fruits when inoculated with *Rhizoglomus fasciculatum* compared with *Glomus macrocarpum*, that modified also the essential oil profiles, with increased concentrations of linalool and geraniol, respectively (Kapoor et al., 2002b). By contrast, the same AMF species produced completely opposite results with dill and carum plants, where *G. macrocarpum* was more efficient in enhancing essential oil concentrations, compared with *R. fasciculatum* (Kapoor et al., 2002a). Experiments with other AMF species confirmed the occurrence of differential activity, as shown by data on the stimulation of root thymol derivative production, which was higher upon inoculation with *Rhizoglomus clarum* in *Inula ensifolia* roots (Zubek et al., 2010), and on the enhancement of shoot hypericin and pseudohypericin contents in *Hypericum perforatum* by *Rhizoglomus intraradices* (Zubek et al., 2012). Single species inocula showed different results also in artichoke, basil, lettuce, pepper and tomato (Table 1). When AMF species other than Glomeraceae were used as inoculum, large variations were found in the concentration of basil leaves essential oils: *Gigaspora rosea* largely increased the concentration of camphor and alfa-terpineol, while *Gigaspora margarita* highly decreased eucalyptol, linalool, eugenol content, and the total content of essential oils (Copetta et al., 2006).

As in agricultural ecosystems many different AMF co-occur in individual plants, it is important to assess plant performance in response to inoculation with multiple AMF species and/or isolates, either originating from natural communities or laboratory assemblages. Only few authors adopted such an approach, revealing the enhancement of some beneficial compounds by AMF mixtures, compared with single-isolate inoculation (Table 1). For example, total phenolic content (TPC) and antioxidant activity increased in leaf extracts and in flower heads of artichoke plants inoculated with a mixed inoculum, compared with single species inocula, both in microcosm and in the field, suggesting a synergistic effect and/or a functional complementarity between the AMF species (Ceccarelli et al., 2010). In another study the antioxidant activity expressed as the ferric reducing ability of plasma assay (FRAP) was enhanced in onion by a mixed commercial inoculum containing *Claroideoglomus etunicatum, Rhizoglomus microaggregatum, R. intraradices, Claroideoglomus claroideum, F. mosseae, Funneliformis geosporus* compared with a single species (*R. intraradices*) inoculum (Albrechtova et al., 2012). The use of mixed inocula vs. single species-inoculum showed in tomato contrasting results, depending on the active molecule involved in the modulation: the mixed AMF species *F. mosseae* BEG12 and *R. irregulare* BB-E increased β-carotene and total carotenoids levels with no effects on lycopene content, while an opposite result was obtained using the two species individually (Hart et al., 2015).

One possible mechanism by which AMF stimulate phytochemical production could be through enhanced nutrient uptake, especially P. Basil plants inoculated with *Funneliformis caledonius* and *F. mosseae* showed higher levels of rosmarinic acid (RA) in the shoots, compared with control plants of the same P status, suggesting that the increased RA concentrations were not exclusively the results of a better P nutrition (Toussaint et al., 2008). However, the effects of an improved N nutrition remain to be investigated, as the higher N assimilation in mycorrhizal plants could contribute to the synthesis of the aminoacids tyrosine and phenylalanine and to a higher production of phenylalanine ammonia-lyase, an enzyme involved in the production of RA (Petersen and Simmonds, 2003).

The parameters to be taken into account in order to select the best performing AMF isolates, species or communities, should refer not only to the content of the health-promoting secondary metabolites, but also to the levels of transcripts encoding the enzymes of the relevant pathways. Only two studies, related to the trascriptome of tomato (López-Ráez et al., 2010b) and grapevine (Balestrini et al., 2017) roots, used different AMF inocula. In tomato, *R. intraradices* was able to positively regulate a larger number of genes related to health bioactive molecules, compared with *F. mosseae*, whereas no differences in the modulation of genes by the two inoculants were observed in grapevine (Table 2). The ability of different AMF species and isolates to regulate genes related to beneficial phytochemicals should be further investigated by wide transcriptome studies, which could also provide insights into P and N nutrition-dependent effects. In particular, in the years to come metabolomic and transcriptomic analyses should be performed in experiments carried out under commercial production conditions—the normal way to grow plants used for human consumption—in order to avoid false positive responses.

## Conclusion and future perspectives

The role played by AMF in the production of health-promoting phytochemicals by host plants has been widely investigated, using multimodal approaches. Although most of the studies showed increases in the levels of several phytochemicals in inoculated plants, some drawbacks hinder the implementation of these beneficial fungi for the production of high quality foods.

One of the main weakness concerning the data obtained so far entails the use of a low number of AMF species (about 24), generally the same ones utilized all over the world, that has limited the full exploitation of their wide physiological and genetic diversity. As the AMF species described so far are about 300, future studies should be performed utilizing the highest possible range of diverse AMF, not only at the species level, but also at the level of isolates and lineages within isolates (Chen et al., 2018; Savary et al., 2018), in order to select the best performing symbionts. Indeed, most of the physiological and functional characteristics of microorganisms, from bacteria to fungi, are properties of each individual strain. Unfortunately, the studies discussed so far often reported just the name of the AMF species utilized, and not always described the isolates, either with their number/name or with the name of the collection where they originated from.

Moreover, some of the studies analyzing the efficiency of laboratory-selected or commercial AMF strains when inoculated in the fields, found that their establishment and persistence were limited, due to the competition with well-adapted indigenous communities (Pellegrino et al., 2012; Loján et al., 2017). Such findings suggest that the selection of native AMF isolates could lead to more successful results. In order to scale up mesocosm experiments, as the success of AMF field inoculation is affected by many factors (Berruti et al., 2016), a more feasible and broad-spectrum approach is represented by nursery inoculation of fruit trees or vegetable plant species before field transplant, which may allow early AMF establishment in roots and field persistence (Ceccarelli et al., 2010; Alaux et al., 2018).

Considering that in nature many different species and strains coexist in the same field, it is crucial to increase studies on the different AMF mixtures for detecting possible synergistic effects and functional complementarities among them, leading to a further selection of the best AMF combinations. Moreover, additional works should be performed on the effects of the combined inoculation of AMF with biostimulants and other beneficial microorganisms, such as plant growth promoting bacteria and fungi (i.e., *Trichoderma* spp.), whose metabolic activities could affect the outcome of the mixed inoculum (Lingua et al., 2013; Colla et al., 2015; Rouphael et al., 2015).

So far, RNA-Seq technology, together with a mechanistic approach, has been utilized for investigating the differential gene expression, mostly in root tissues. Further transcriptomic studies should explore the differential expression of genes involved in the biosynthesis of health-promoting plant compounds in edible tissues of mycorrhizal plants. Such a powerful technology should be applied in order to unravel the mechanisms encompassing the enhancement of health-promoting phytochemicals biosynthesis, as affected by the widest possible range of AMF. This will increase our knowledge on the specific role played by each strain, in order to utilize sound criteria for a finely tuned, targeted selection of the best performing symbionts, to be used as sustainable biotechnological tools for the production of safe and healthy plant foods.

## Author contributions

All authors listed have made a substantial, direct and intellectual contribution to the work, and approved it for publication.

### Conflict of interest statement

The authors declare that the research was conducted in the absence of any commercial or financial relationships that could be construed as a potential conflict of interest.
